# Molecular Identification and Characterisation of a Spiro-Indoline-Benzoxadiazine Derivative for Photochromic Textile Sensors

**DOI:** 10.3390/ijms27114704

**Published:** 2026-05-23

**Authors:** Elżbieta Sąsiadek-Andrzejczak, Malwina Jaszczak-Kuligowska, Marta Safandowska, Marek Kozicki, Bożena Rokita, Laura Florentino-Madiedo, Marcin Barburski, David Ranz, Reyes Mallada

**Affiliations:** 1 Department of Mechanical Engineering, Informatics and Chemistry of Polymer Materials, Faculty of Textiles and Design, Lodz University of Technology, Zeromskiego 116, 90-543 Lodz, Poland; malwina.jaszczak@p.lodz.pl (M.J.-K.); marek.kozicki@p.lodz.pl (M.K.); 2 Centre of Molecular and Macromolecular Studies, Polish Academy of Science, Sienkiewicza 112, 90-363 Lodz, Poland; marta.safandowska@cbmm.lodz.pl; 3 Institute of Applied Radiation Chemistry, Faculty of Chemistry, Lodz University of Technology, Wroblewskiego 15, 93-590 Lodz, Poland; bozena.rokita@p.lodz.pl; 4 Instituto de Nanociencia y Materiales de Aragón, Universidad de Zaragoza, C/Mariano Esquillor, s/n Ca pus Río Ebro, 50018 Zaragoza, Spain; lflorentino@unizar.es (L.F.-M.); rmallada@unizar.es (R.M.); 5 Institute of Architecture of Textiles, Faculty of Textiles and Design, Lodz University of Technology, Zeromskiego 116, 90-543 Lodz, Poland; marcin.barburski@p.lodz.pl; 6 Departamento de Ingeniería de Diseño y Fabricación, Universidad de Zaragoza, María Luna 3, 50018 Zaragoza, Spain; dranz@unizar.es

**Keywords:** photochromic sensors, photochromic pigment, screen-printed textiles, UV radiation sensor, textile sensor

## Abstract

This paper describes the comprehensive molecular characterisation and application of a commercially available, but structurally undefined, photochromic pigment for the development of textile sensors. The commercial pigment was successfully identified using a multianalytical approach, including analysis using nuclear magnetic resonance (NMR), Fourier transform infrared spectroscopy (FTIR), and differential scanning calorimetry (DSC). The identified pigment, ethyl-3′-methyl-3′-phenyl-1′-(propan-2-yl)-1′,3′-dihydrospiro[[4,1,2]benzoxadiazine-3,2′-indole], was used to develop a textile sensor by screen printing on a natural fibre fabric surface. The developed sensor exhibited a reversible colour change from white to pink upon exposure to UVA radiation (369 nm). The sensor is characterised by high sensitivity with a linear dose–response of 0–0.005 J/cm^2^ and a dynamic range of up to 0.05 J/cm^2^. Furthermore, the sensor’s molecular safety profile was assessed, including elemental composition and cytotoxicity tests on human dermal fibroblasts, which confirmed the sensor’s biocompatibility with occasional skin contact. In addition to its use in decorative and security elements for product authentication, this study demonstrates the sensor’s ability to map the 2D UVA radiation dose distribution. This research highlights the importance of precise molecular identification in the design of functional, safe, and intelligent textile systems.

## 1. Introduction

Photochromic dyes and pigments are chemical compounds that change colours under the influence of ultraviolet radiation from the sun and artificial sources. The colour change is a reversible or non-reversible chemical reaction that alters molecular structure in response to the presence or absence of UV light. The photochromic compounds revert to their original colour upon removal of the light source. In general, the photochromic reaction mechanism involves a reversible molecular change between two isomers of a compound, one of which is coloured, and the other is not. When UV radiation strikes the photochromic compound, it causes a molecular change that alters the way the colourant absorbs and reflects visible light, resulting in a colour change [1,2]. Depending on the ability of the compound to undergo reversible colour changes when exposed to UV radiation, a distinction is made between: (i) organic photochromic dyes and pigments, which undergo reversible structural changes when exposed to UV radiation, including spiropyrans, spirooxazines, naphthopyrans, fulgides, and fulgimides [3,4], (ii) inorganic photochromic compounds, which are derivatives of metal oxides [5], (iii) hybrid materials, which are a combination of photochromic molecules into an inorganic matrix [6], (iv) chromene derivatives, which exhibit faster colour transitions and excellent fatigue resistance [7], and (v) viologens, which are organic salts that can undergo reversible redox reactions when exposed to UV radiation [8].

The origin of the photochromic compound and its structure determine its use in various areas of material functionalisation, including (i) optical systems, especially in the production of lenses for photography and glasses [9], (ii) the production of paints and inks [10], (iii) UV radiation sensors and smart materials [11], and (iv) textiles [3,12,13,14]. The automotive industry [15], medicine [16], and modern urban architecture commonly utilise solutions like self-darkening windows and packaging [17,18,19] exposed to UV radiation. Among the publications, spirooxazines are well described, e.g., 1,3,3-trimethyl-6′-(piperidin-1-yl)spiro[indoline-2,3′-naphtho [2,1-b][1,4]oxazine]. This photochromic pigment consists of a spiro-structure with an indoline component attached to an oxazine group. The photochromic functionality of pigment is dependent on the oxazine group. Under the influence of UV radiation, the closed, colourless form of the molecule undergoes a structural change in which the spiro bond breaks, opening the oxazine ring and creating a new, extended π-conjugated system. Once the exposition to UV radiation is finished, the molecule returns to its original colourless, closed ring form. The colour change of this pigment is rapid, with the UV-activated form appearing within seconds of exposure and then gradually reverting to colourless after UV exposure ends. The rate of fading of this pigment can be controlled by the temperature of the surrounding medium or its chemical composition. The biggest disadvantage is the material fatigue effect, which reduces the effectiveness of the colour change over time and after prolonged exposure to visible light, including sunlight, artificial sources of UV radiation, and heat [20].

The textile industry utilizes various types of textile modification, including surface modification (such as dyeing, printing, and coating) and volume modification (such as dyeing fibres by incorporating a colorant into the polymer mass). Modification of fibres with UV-sensitive compounds is very interesting and described in the literature, e.g., polyacrylonitrile fibres modified with 2,3,5-triphenyltetrazolium chloride (TTC) and 4,4′,4″-tri-di-β-hydroxyethylaminotriphenylacetonitrile (HHEVC) can be used as UV radiation sensors in a wide dose range up to 10 J/cm^2^ [21]. A fabric made of polyacrylonitrile fibres doped with 10,12-Pentacosadiynoic acid (PENTA) can be used as a dosimeter to measure the 2D dose distribution of ionising radiation in the dose range of up to 15 kGy [22]. However, this work was limited to surface methods of textile modification. Dyed textiles can be produced by using (i) dyeing with dyes for natural and synthetic materials and their mixtures, (ii) dyeing with pigments, mainly for synthetic materials, and iii) printing for natural and synthetic materials and their mixtures [21,23]. Dyes and pigments are often used in textile printing. Usually, they are combined with printing pastes containing a thickener and a binder. After applying a thin layer to a surface, for example, fabric, the paste is usually thermally fixed in the drying and heating process. Depending on the chemicals used, the coating produced can be stiff or elastic, which allows the modified textile material to remain flexible. In comparison to traditional textile dyeing methods, the printing method using water-based printing pastes is fast, cheap, and does not require large amounts of water and chemical auxiliaries [24]. Furthermore, solutions for printing surfaces of materials such as cotton and polyamide using colour precursors to assess the doses and distribution of ionising and UV radiation are well known. For example, systems based on polyamide or cotton fabric and a printing paste containing nitro blue tetrazolium chloride (NBT) [25], 2,3,5-triphenyltetrazolium chloride (TTC), leuco crystal violet (LCV), or leuco malachite green (LMG) [26,27,28] can be used for UVA, UVB, and UVC radiation dose assessments in dose ranges up to 10 J/cm^2^. It has been shown that the use of radiation-sensitive compounds as printed layers on the surface of textile products can be used as UV radiation dosimeters [25,26,27,28,29,30]. Radiochromic colour precursors, under the influence of UV irradiation, changed their colour from colourless to blue, red, and green. The colour change is not reversible, and the intensity increases as the absorbed UV radiation increases. Colour changes can be evaluated using a reflectance spectrophotometer, which allows us to obtain information about the colour of dosimeters and the accurate characterisation of the basic properties of such dosimeters, including: (i) dose sensitivity; (ii) linear and dynamic dose–response; and (iii) threshold dose. Moreover, such dosimeters can be used for (i) personal protection against UV radiation; (ii) marking textile products; (iii) protecting textiles and paper against counterfeiting; and (iv) having a decorative design role.

The aim of this work was to develop a reusable, flexible, photosensitive UV radiation sensor using screen printing with a printing paste containing a photochromic pigment. Due to the commercial nature of the compound used in the study, whose chemical structure is unknown and protected by the manufacturer, the main part of the research was focused on analysing the chemical composition of the photochromic pigment using nuclear magnetic resonance spectroscopy (NMR), Fourier transform infrared spectroscopy (FTIR), differential scanning calorimetry (DSC), elemental analysis, and scanning electron microscopy (SEM). Unlike commercially available textile materials modified with photochromic compounds, the developed sensor was described in detail in terms of its fabrication method and characterisation in response to UV radiation. It was assumed that the developed sensor: (i) takes the form of a layer printed on the surface of a flat textile product; (ii) is flexible; (iii) provides information on the absorbed UV radiation dose, which is the total dose of UVA (95%) and UVB (5%) radiation; (iv) has reversible colour change; (v) can be compared to an accepted calibration scale; and (vi) can be used as a standalone sensor or incorporated into composite products. Additionally, it was demonstrated that the developed sensor can be used to assess UV radiation dose distribution, which is a novelty in this field. The printed sensor was characterised in terms of the uniformity of the layer produced on the textile surface, response to UV radiation dose, stability over time, and repeatability of spectrophotometer measurements. Methods for using the developed sensors in personal protective equipment, packaging, and decorative elements were proposed.

## 2. Results and Discussion

### 2.1. Chemical Analysis of Photochromic Pigment

In the first stage of chemical analysis, the purchased pigment was subjected to solubility tests, which were carried out using various solvents: water, methanol, ethanol, acetone, toluene, isopropyl alcohol, pyridine, and dimethylformamide (DMF). For this purpose, 1 mg of colourant was mixed with 10 mL of pure solvent using a magnetic stirrer. After 120 min of continuous mixing at a rotation speed of 250 rpm, the mixtures were assessed organoleptically up to 7 days after their preparation. In the case of acetone and DMF, the mixture was heated to 60 °C. In none of the variants was the added colourant observed to dissolve. In all variants, a white turbid precipitate remained, which still changed colour to pink after UV irradiation, except for acetone and hot DMF. From data from other manufacturers of similar chemical compounds, e.g., QCR Solutions Corp., it appears that these may also be photochromic microencapsulated dyes, which are specially designed for use in non-aqueous-based ink systems, and they can be used to formulate non-aqueous-based flexographic, UV, screen, offset, gravure, and epoxy ink formulations. However, analysing the results obtained for the solvents used, it was decided to maintain the conclusion that the purchased chemical compound is a photochromic pigment.

**Figure 1:**
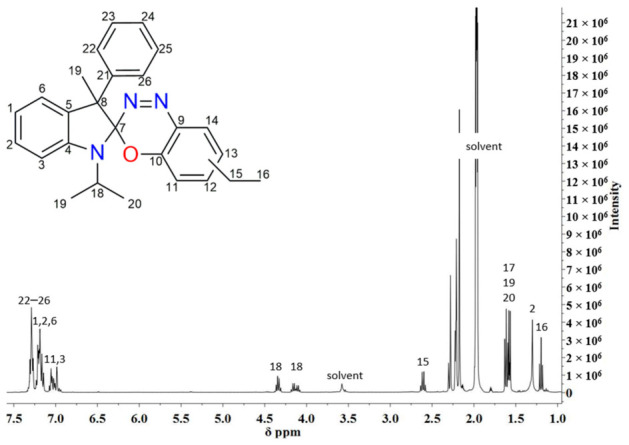
^1^H NMR spectrum of the photochromic pigment (400 MHz, CD_3_CN).

**Figure 2:**
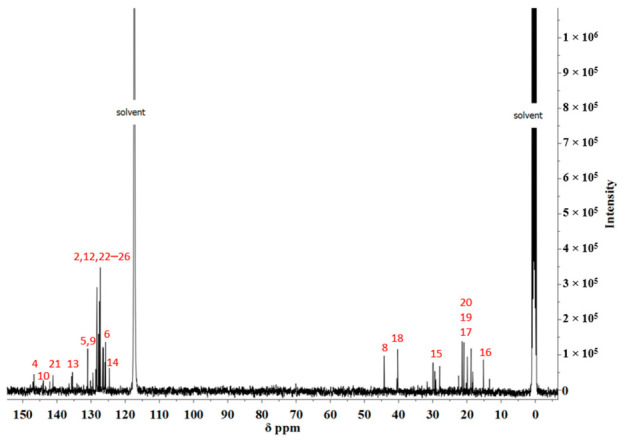
^13^C NMR spectrum of the photochromic pigment (400 MHz, CD_3_CN).

To determine the chemical composition of the compound purchased for research, an analysis was performed using spectroscopic methods. Figure 1 and Figure 2 depict the representative ^1^H NMR and ^13^C NMR spectra, with peak assignments labelled accordingly in the structural formulas. In the ^1^H NMR spectrum (400 Hz, CD_3_CN) of the analysed pigment, distinct signals were detected at the following chemical shifts: δ (ppm) = 1.20 (3H, t, 12-CH_2_-CH_3_), 1.30 (3H, s, 8-CH_3_), 1.55–1.64 (6H, m, -N-CH-(CH_3_)_2_), 2.61 (2H, q, 13-CH_2_-CH_3_), 4.35 (1H, p, -N-CH-(CH_3_)_2_), 6.92–7.10 (3H, m, 11-H, 1-H, 3-H), 7.13–7.23 (4H, m, 24-H, 26-H, 2-H, 14-H), 7.25–7.34 (5H, m, 8-C_6_H_5_) (Figure 1). The ^13^C NMR spectrum (400 Hz, CD_3_CN) of the photochromic pigment revealed signals with chemical shifts: δ (ppm) = 15.19 (1C, 12-CH_2_-CH_3_), 18.80–20.90 (2C, -N-CH-(CH_3_)_2_), 21.43 (1C, 8-CH_3_), 27.99–29.9 (1C, 13-CH_2_-CH_3_), 40.5 (1C, -N-CH-(CH_3_)_2_), 44.2 (1C, 8-Cq), 124.6 (1C, 9-C-CH=), 125.8 (1C, 5C=CH-CH=), 126.5–127.8 (5C, 8-C_6_H_5_; 1C,1-CH=CH-CH=; 1C, 11-CH=CH-CH=), 128.2 (1C, 6-CH=C-C; 1C =N-C-CH=), 131.0 (1C, 14-CH=C-CH_2_-), 135.4 (1C, 8-C_6_H_5_), 141.1 (1C, 7-O-C=C), 146.7 (1C, 5-CH=C-N) (Figure 2).

**Figure 3:**
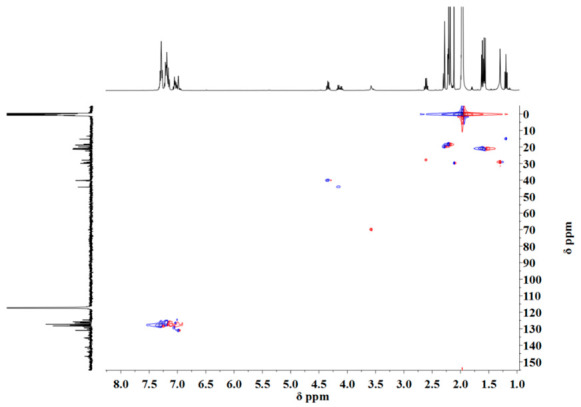
2D HSQC NMR spectrum of the photochromic pigment (400 MHz, CD_3_CN).

**Figure 4:**
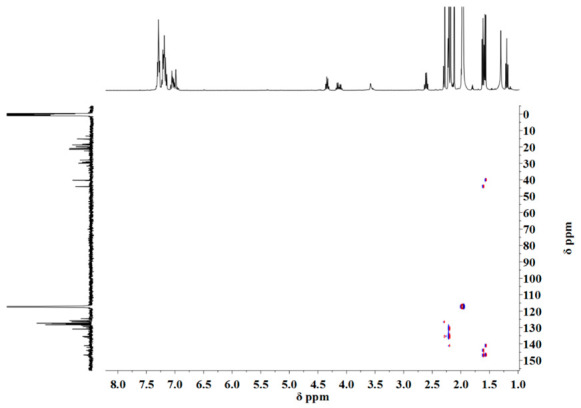
2D HMBC NMR spectrum of the photochromic pigment (400 MHz, CD_3_CN).

**Figure 5:**
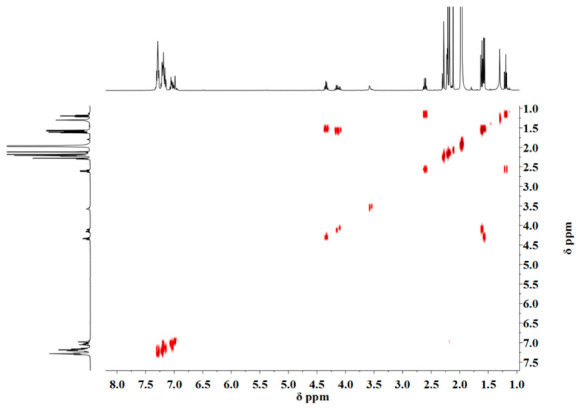
2D COSY NMR spectrum of the photochromic pigment (400 MHz, CD_3_CN).

The assignment of proton signals in the ^1^H NMR spectrum was supported through the analysis of two-dimensional NMR techniques. The HSQC and HMBC spectra provided information on direct and long-range correlations between protons and corresponding carbon atoms (Figure 3 and Figure 4). COSY correlates the chemical shifts of spins that share a mutual J-coupling and was utilised to investigate couplings between protons (Figure 5). All signals that appear in the one-dimensional spectrum will show a peak along the diagonal in the COSY spectrum. The cross-peaks (off-diagonal peaks) show which hydrogens share a J-coupling through the correlation between the two chemical shifts [31,32]. The cross-peaks observed in the spectrum provided valuable information on proton connectivity and facilitated the identification of distinct spin systems within the pigment structure. The COSY spectra confirmed scalar couplings within the ethyl and isopropyl groups and facilitated identification of proton spin systems in the aromatic region. The 2D NMR experiments allowed improved differentiation of partially overlapping aromatic signals observed in the downfield region of the ^1^H NMR spectrum. The combined analysis of ^1^H, ^13^C, HSQC, HMBC, and COSY spectra enabled the proposed structural interpretation of the investigated photochromic pigment. Based on literature data concerning aromatic substitution patterns and characteristic functional groups of photochromic compounds, together with spectral predictions generated using Mnova 15.1.0 software for various photochromic systems, the pigment was assigned to an ethyl-3′-methyl-3′-phenyl-1′-(propan-2-yl)-1′,3′-dihydrospiro[[4,1,2]benzoxadiazine-3,2′-indole]-type structure [33,34,35,36].

Elemental composition analysis (CHNS) confirmed the presence of carbon (77.23%), hydrogen (7.42%), nitrogen (9.75%), and oxygen (5.60%) in the photochromic pigment. These results align with the theoretical values (C: 78.56%; H: 6.85%; N: 10.57%; O: 4.02%) for structurally related compounds, such as ethyl-3′-methyl-3′-phenyl-1′-(propan-2-yl)-1′,3′-dihydrospiro[[4,1,2]benzoxadiazine-3,2′-indole] with the molecular formula C_26_H_27_N_3_O and average mass 397.51 g mol^−1^. A slight discrepancy in the above values may be related to the presence of a dispersant additive in the photochromic pigment, which is also indicated by the overlapping signals in some areas in the NMR spectra.

**Figure 6:**
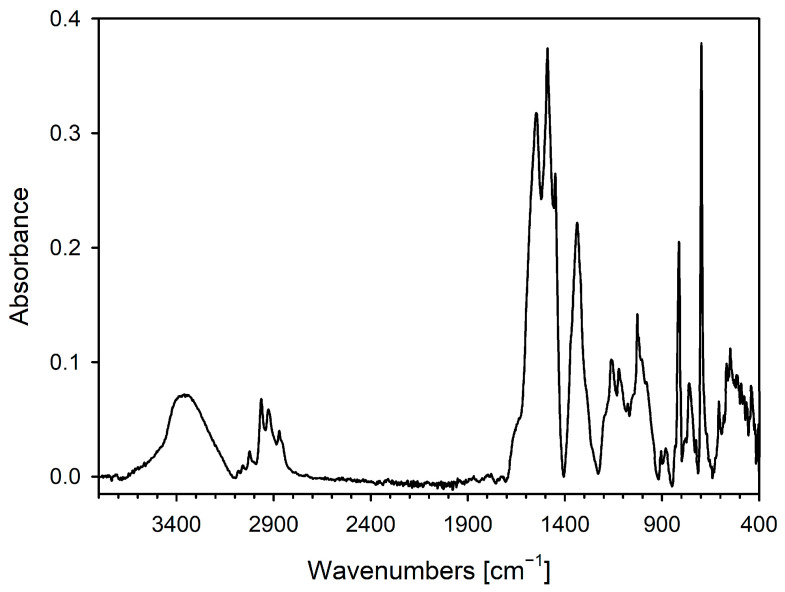
Fourier transform infrared (FTIR) spectra of photochromic pigment.

The chemical bonding structure was obtained from FTIR-ATR spectroscopy. Figure 6 shows the FTIR spectrum of the analysed photochromic pigment. In the spectra, the peak at 3359 cm^−1^ contributes to the absorption of O-H stretching vibration and indicates the presence of water in the pigment [37]. In this wavenumber range, both O-H stretches and N-H stretches can occur; however, in our case, N-H bonds are not present. The O-H bonds are more polar than N-H bonds, therefore form stronger hydrogen bonds, and hence have wider peaks than N-H bonds. The analysed spectrum contains several peaks confirming the presence of unsaturated aromatic rings [38,39]. The peaks in the region of 3100–3000 cm^−1^ can be attributed to C-H stretching vibrations, the peaks from 1600 to 1400 cm^−1^ are ring modes (C-C stretching vibrations in the ring), while the peaks at 812 cm^−1^ and 697 cm^−1^ indicate benzene ring substitution (aromatic C-H wagging) and the presence of ring bend, respectively. The absorption peaks at 2964, 2926 and 2869 cm^−1^ are the CH_3_ asymmetric C-H stretch, the CH_2_ asymmetric stretch, and the CH_3_ symmetric stretch, respectively. The FTIR spectrum exhibits also distinct absorption band at 1336 cm^−1^ including C-N stretching vibration present in the spirobenzoxadiazine molecule [33,40]. The bands in the region of 1200–900 cm^−1^ are typically assigned to C-O stretching vibrations of aromatics. To sum up, the absorption peaks visible in the FTIR spectrum indicate that the analysed photochromic pigment belongs to the group of spirobenzooxadiazine [33] and at the same time confirm that it may be a compound with the molecular formula C_26_H_27_N_3_O.

**Figure 7:**
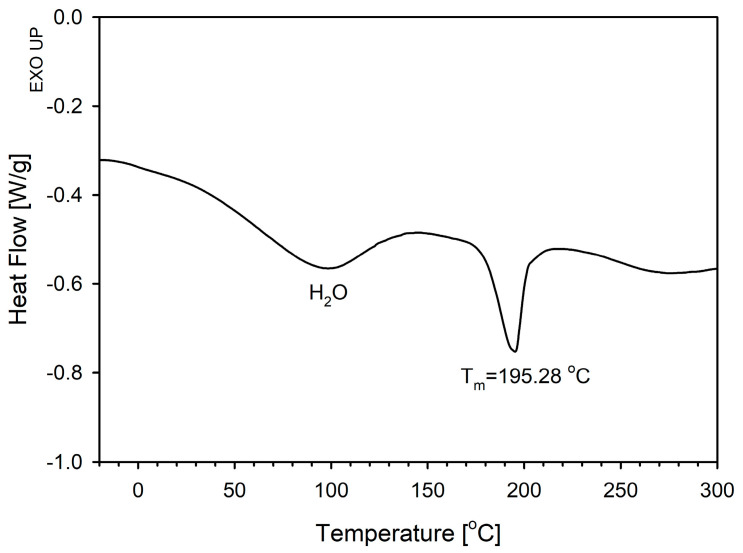
Differential scanning calorimetry (DSC) thermogram of photochromic pigment.

Additionally, differential scanning calorimetry (DSC) analysis was performed for the examined pigment. Figure 7 shows the endothermic peaks from the first heating curves of the photochromic pigment. From the DSC thermogram, it can be observed that samples of photochromic pigment display two thermal transitions: water evaporation from the sample (T = 94.70 °C) and the melting temperature (T_m_ = 195.28 °C) of photochromic pigment. During the analysis, it was confirmed that the NMR spectra obtained for the purchased photochromic pigment do not coincide with the spectrum of the compound declared by the manufacturer and described by CAS number 114747-45-4 (1,3,3-Trimethylindoline-6′-(1-piperidinyl)spironaphthoxazine) in the technical specification. Based on the conducted spectroscopic analysis, elemental composition analysis, thermal properties, and multiple literature sources [34,41,42,43,44,45], it can be concluded that the analysed pigment does not belong to the spiropyran or spirooxazine group. In addition, differences between theoretical and measured values in the CHNS analysis (e.g., higher hydrogen and oxygen content compared to the model) are typical for technical pigments containing hygroscopic additives and organic excipients (e.g., dispersants and surfactants), as confirmed by the presence of a water signal in the FTIR spectrum (3359 cm^−1^) and an endothermic peak in the DSC curve (94.70 °C).

**Figure 8:**
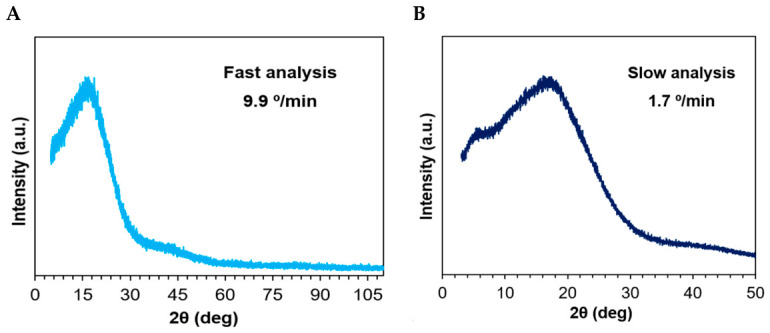
XRD patterns of photochromic pigment scanned at two 2θ range and scanning rate: (**A**) 5–110°, 9.9°/min; (**B**) 3–50°, 1.7°/min.

In parallel to the NMR studies, X-ray diffraction (XRD) analysis was conducted to investigate the morphological structure of the tested photochromic pigment. Two measurements were performed at different scan rates: 9.9°/min and 1.7°/min. The slower analysis was performed to increase the measurement resolution and determine a better signal-to-noise ratio. In both cases, broad diffraction peaks were observed in the 2θ range of 10–30°, with the maximum intensity around 20 degrees (Figure 8A,B). Due to the lack of sharp diffraction peaks and notable differences between the two XRD plots, the previous assumptions that the tested pigment is amorphous material were confirmed.

**Figure 9:**
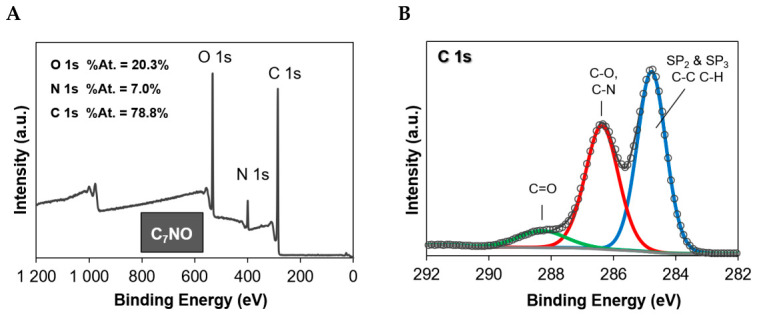

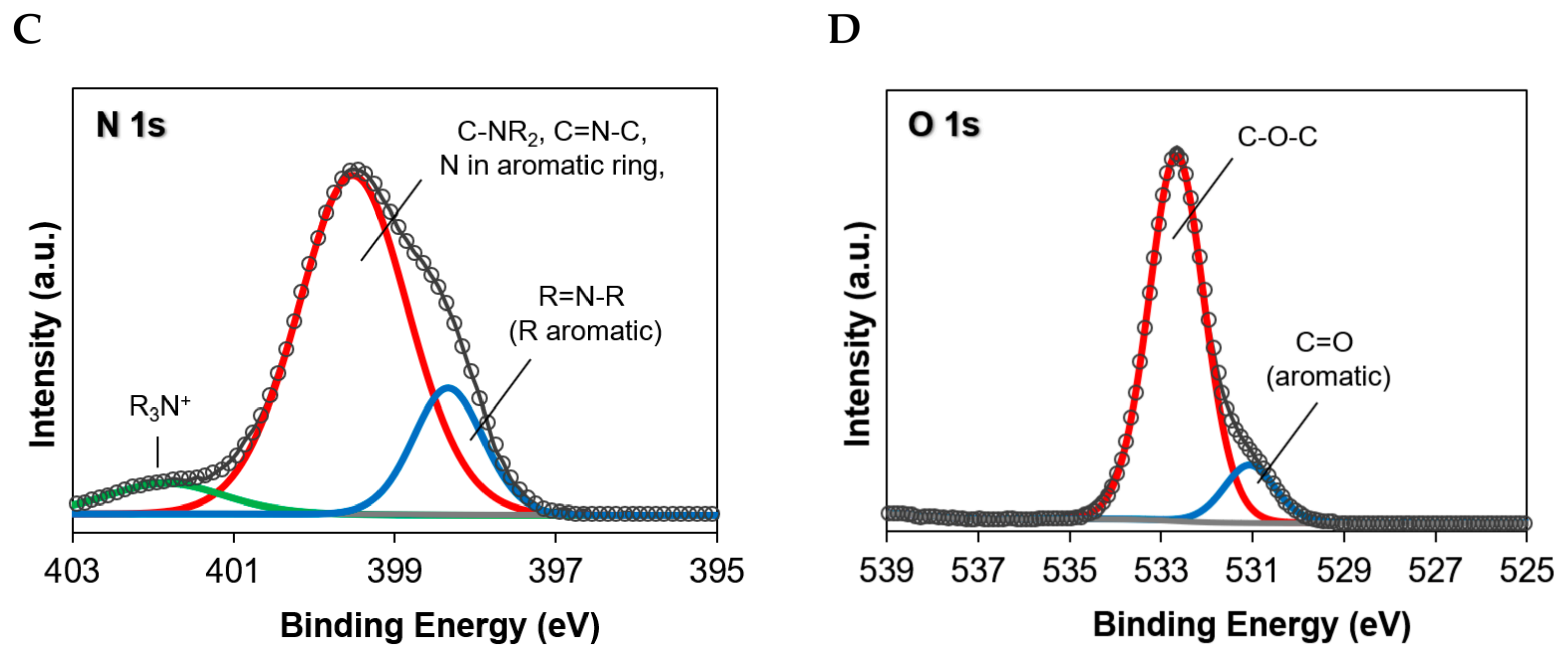
XPS spectra of photochromic pigments: (**A**) broad scan; (**B**) deconvoluted carbon 1s spectra; (**C**) deconvoluted nitrogen 1s spectra; and (**D**) deconvoluted oxygen 1s spectra.

Additionally, XPS analysis was performed, which showed the general elemental composition of the analysed sample. Three main peaks visible in Figure 9A correspond to carbon (peak around 285 eV), nitrogen (peak around 400 eV), and oxygen (peak around 532 eV). Based on the obtained results, the percentage share of individual elements was also determined, which was 7.0%, 20.3%, and 78.8% for nitrogen, oxygen, and carbon, respectively. Deconvolution spectra for high-resolution C 1s, N 1s, and O 1s spectra are shown in Figure 9B–D. In the case of carbon spectrum analysis (Figure 9B), it was shown that there are three different types of carbon bonds in the sample: (i) carbon in sp^2^ hybridisation (band at 284.8 eV) with low bond energy, which usually occurs in aramid or double C=C rings, and carbon in sp^3^ hybridisation (band at 286.3 eV) characteristic of aliphatic chains or single C-C and C-H bonds; (ii) carbon bonds with oxygen or nitrogen (band at 286.3 eV) with higher bond energy, which are characteristic of alcohols, ethers (C-O), or amines (C-N); and (iii) carbonyl bonds (band at 288.6 eV) with the highest C=O bond energy, which are characteristic of aldehydes, ketones, or carboxylic acids. In the case of nitrogen spectrum analysis (Figure 9C), it was shown that the molecule has two types of nitrogen bonds in the sample: (i) nitrogen in the aromatic ring (band at 399.3 eV) or bonded to carbon in the form of C-NR_2_ (characteristic bond for secondary amines) and C=N-C (characteristic bond for imine); and (ii) nitrogen in azo bonds with aromatic groups R=N-R (band at 398.2 eV). Additionally, it was shown that in the band at 401.7 eV there are also bonds with higher bond energy, suggesting the occurrence of the R_3_N^+^ group, which are characteristic of ammonium salts or are formed as a result of protonation of amine groups. Analysing the oxygen spectrum (Figure 9D), two types of bonds were observed in the molecule: (i) oxygen bonds in single bonds with carbon atoms (band at 533.0 eV) characteristic of ether groups and alcohols; and (ii) oxygen bonds in carbonyl groups C=O (band at about 531.6 eV), which is characteristic of systems associated with an aromatic ring, e.g., in quinones and carboxylic acids. Thus, the chemical analysis performed confirmed the occurrence of characteristic photochromic pigment bonds. The presence of bands corresponding to carbonyl groups (C=O) in the XPS spectrum, in the absence of these in the structure of the main chromophore, is attributed to the presence of carrier resins or pigment coating substances (microencapsulation), which is consistent with the information about the pigment’s intended use in non-aqueous ink systems (flexo, offset, UV). Based on the assumptions, it was possible to establish a model of the molecule, although its exact structural configuration remains uncertain. This ambiguity may be attributed to the fact that the purchased compound is a synthetic pigment, likely developed through the partial combination of various molecular fragments. However, further investigation into its detailed structure goes beyond the scope of the presented work.

### 2.2. Cytotoxicity

**Figure 10:**
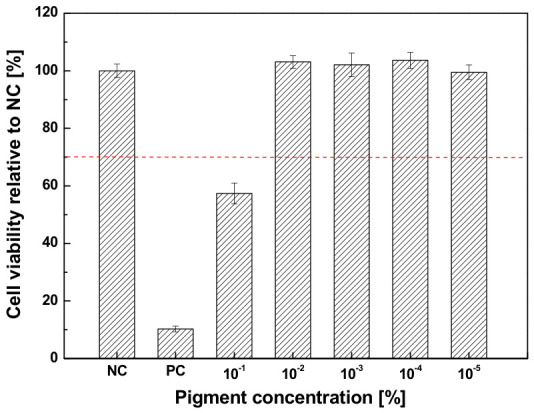
Percentage of cell viability relative to the negative (NC) and positive (PC) control after 48 h culture of human fibroblasts in contact with photochromic pigment suspensions at concentrations of 0.1% to 0.00001%. The red dashed line indicates the cytotoxicity threshold.

A cytotoxicity assessment was conducted to evaluate whether the tested photochromic pigment poses a risk to human skin cells, particularly primary dermal fibroblasts, which play a key role in maintaining skin structure and function. The chemical composition and toxicological profile of such pigments are often poorly characterised, raising concerns about their potential effects on human health and the environment. Considering the possible use of photochromic pigments in skin-contact materials, such as textiles or wearable products, a biocompatibility assessment following the ISO 10993-5 standard was employed [46]. Although this standard is primarily used for medical device evaluation, it also provides a relevant framework for assessing cytotoxicity in other applications where direct skin contact may occur. This approach ensures a high level of safety assessment at an early stage of product development and supports informed decisions regarding the pigment’s suitability for future commercial use. The cytotoxicity analysis of the photochromic pigment in suspension revealed a concentration-dependent effect on cell viability (Figure 10). The highest tested concentration (0.1% *w*/*v*) resulted in a reduction of fibroblast viability to 57.36%, which, according to ISO 10993-5 criteria [46], indicates a cytotoxic response (threshold for cytotoxicity <70% viability relative to control). This suggests that at high concentrations, the pigment suspension may adversely affect cell viability under the applied in vitro conditions. However, the exact mechanism responsible for the observed effect was not investigated in the present study. In contrast, all lower concentrations (ranging from 0.01% to 0.00001%) maintained cell viability at levels comparable to the untreated control (close to 100%), indicating an absence of cytotoxicity. These results indicate that lower pigment concentrations did not induce cytotoxic effects under the applied experimental conditions, whereas higher concentrations should be interpreted with caution.

**Figure 11:**
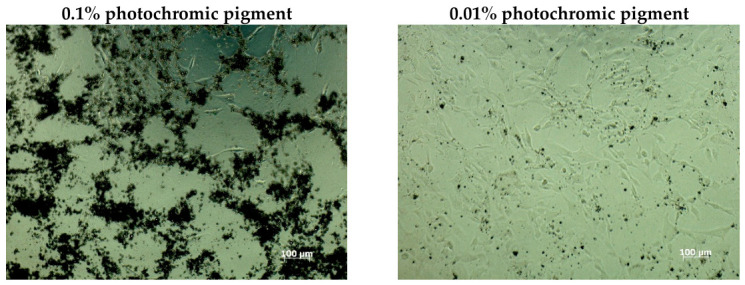

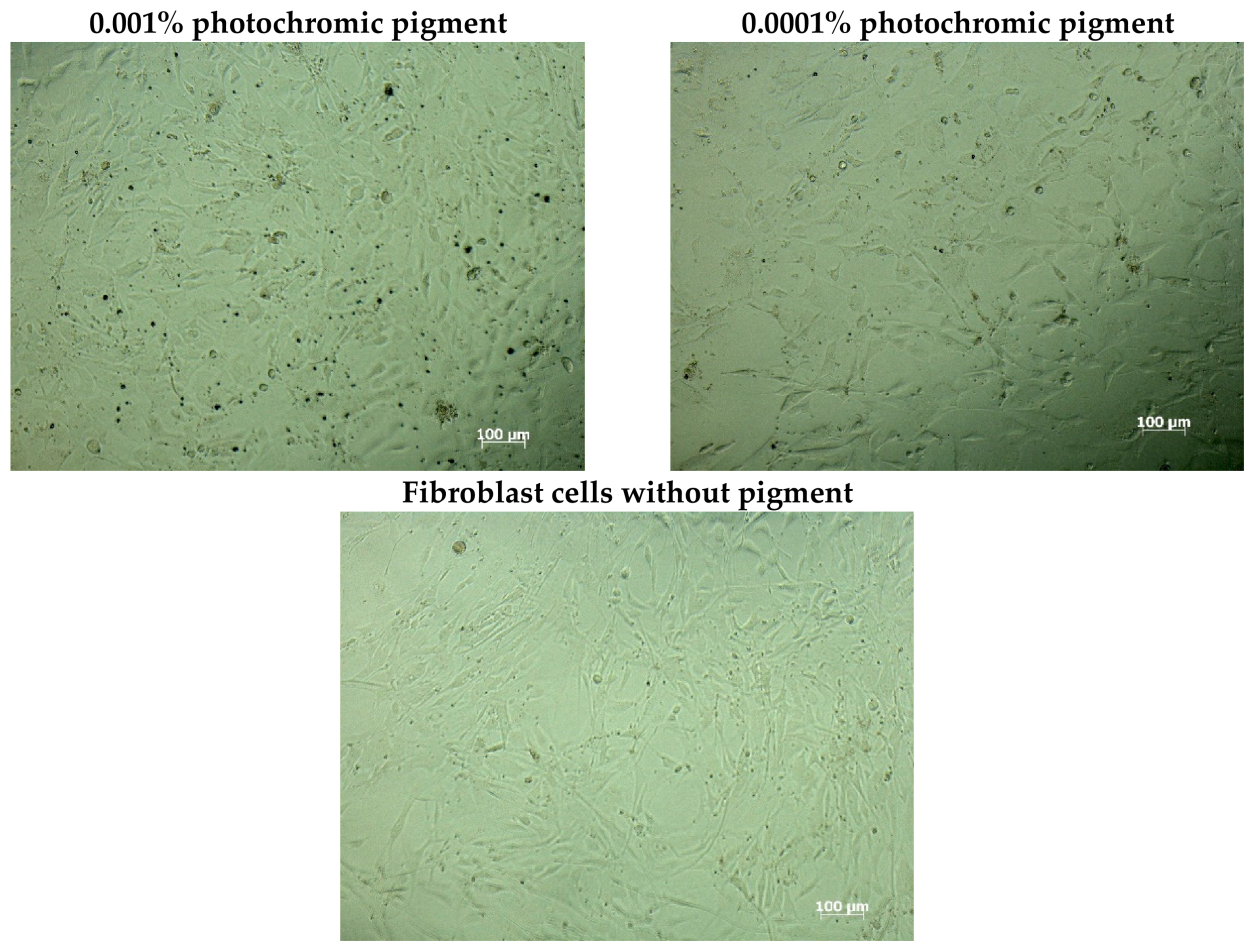
Images of fibroblast cell cultures after 48 h exposure to the test material. Four highest concentrations of unfiltered photochromic pigment.

**Figure 12:**
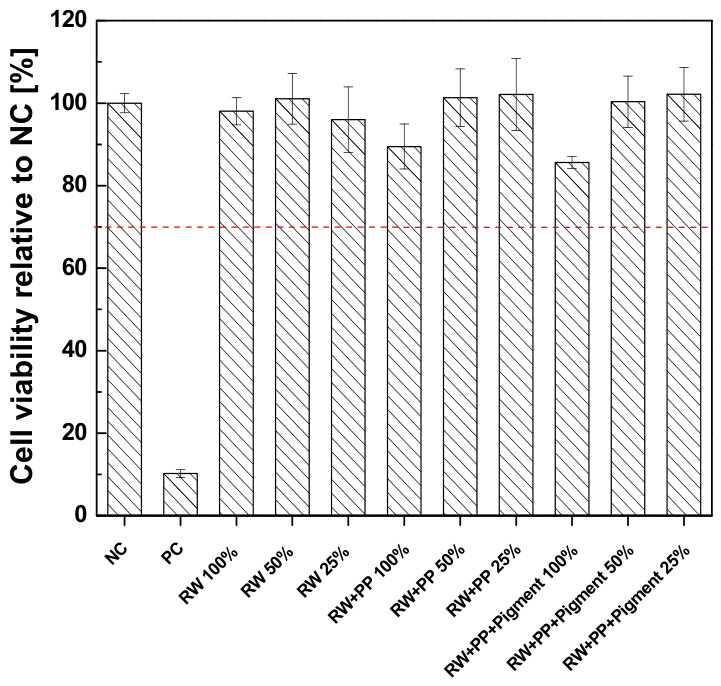
Percentage of cell viability relative to the negative (NC) and positive (PC) control after 48 h culture of human fibroblasts in contact with eluates of the test materials at concentrations of 100%, 50%, and 25%—raw wool (RW), wool printed with plain paste (RW+PP), and wool printed with paste containing photochromic pigment (RW+PP+Pigment). The red dashed line indicates the cytotoxicity threshold.

This concentration-dependent behaviour highlights the importance of concentration selection in product development and underlines the need for proper dispersion protocols and formulation strategies to minimise potential cytotoxic effects. Microscopic observations in transmitted light provided additional insight into the behaviour of the pigment in vitro (Figure 11). At 0.1% concentration, visible pigment particles sedimented onto the bottom of the well, forming a dense layer directly over the cells. These aggregates appeared to physically cover and surround fibroblasts, possibly limiting their access to nutrients or mechanically restricting their ability to proliferate. While fewer cells were present compared to the untreated control, individual cells retained typical morphology, they appeared well-spread and structurally intact, suggesting that the pigment does not induce direct cell lysis but may interfere with cell division at high concentrations. As the pigment concentration decreased, the number and distribution of cells improved, and only sparse pigment particles were observed. The fibroblasts appeared both morphologically healthy and more numerous, indicating the restoration of normal proliferation. These findings indicate that the observed reduction in cell viability at the highest tested concentration may be associated with both direct interactions between the pigment suspension and the cells, as well as physical interactions caused by sedimented particles covering the cell layer. Importantly, exposure conditions corresponding to prolonged direct contact of skin cells with high concentrations of free pigment particles are not expected under realistic use conditions of the printed textile material. In the next stage of the study, the cytotoxic potential of eluates obtained from textile materials was evaluated. The test included eluates from raw wool, wool printed with plain paste (without pigment), and wool printed with the photochromic pigment. Eluates were prepared according to ISO 10993-12 [47] and applied to human dermal fibroblasts at 100%, 50%, and 25% concentrations. Culture medium served as the negative control (Figure 12). Across all samples and dilutions, cell viability remained above the ISO 10993-5 [46] cytotoxicity threshold of 70%, indicating no cytotoxic effects. Eluates from raw wool showed high biocompatibility, with cell viability values of 98%, 101%, and 96% for 100%, 50%, and 25% eluate concentrations, respectively. Similarly, wool printed with plain paste yielded viability values of 89.5% (100%), 101% (50%), and 102% (25%), suggesting that the printing formulation itself does not adversely affect fibroblast viability.

The eluates from wool printed with photochromic pigment showed slightly reduced viability at the highest concentration (85.6% for 100% eluate), but values at 50% and 25% dilutions were 100% and 102%, respectively, comparable to the control and to the paste-only formulation. This indicates that the presence of the photochromic pigment does not significantly increase cytotoxicity compared to the paste alone, even in undiluted eluates. Taken together, these findings demonstrate that none of the textile samples released cytotoxic substances under the extraction conditions used. The pigment, when incorporated into the textile matrix, did not release biologically relevant amounts of potentially harmful compounds under the applied extraction conditions. Thus, the obtained results suggest limited cytotoxic potential of the printed textile system under the applied experimental conditions. Overall, the results demonstrated a concentration-dependent reduction in cell viability in direct pigment suspension tests, whereas eluates obtained from pigment-printed textiles did not induce cytotoxic effects under the applied extraction conditions. The observed reduction in cell viability at the highest tested concentration may have resulted from both the presence of sedimented pigment particles and direct interactions between the pigment suspension and the cells. Importantly, this concentration represents a stringent in vitro exposure scenario that is not expected under normal conditions of textile use. Furthermore, eluates from pigment-printed textiles did not induce cytotoxic effects at any tested dilution, suggesting limited release of biologically relevant amounts of potentially harmful compounds from the printed material. These findings support the further investigation of the developed printed textile system for applications involving occasional or indirect skin contact.

### 2.3. Calibration and Dose–Response to UV Radiation of Printed Wool Sensors

**Figure 13:**
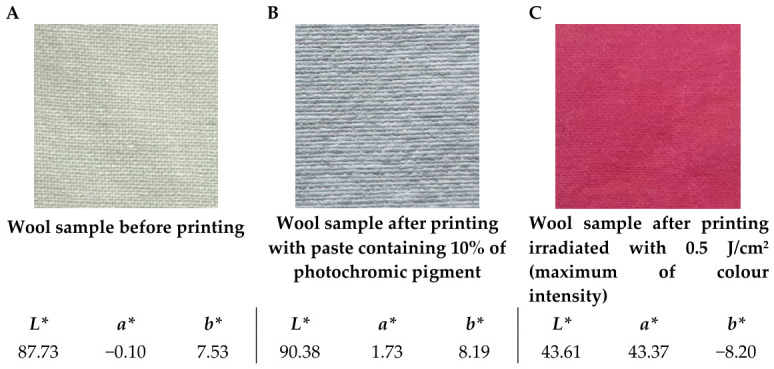
Comparison of wool samples (**A**) before printing; (**B**) after printing with a paste containing 10% photochromic pigment; and (**C**) after UV irradiation with CIE L*a*b* values. The measurement error is 0.1%. All photographs were taken with a Canon 50D digital camera (kit lens: EF-S 18–200 mm f/3.5–5.6; resolution: 15.1 MPix; pixel size: 4.69 µm; standard D65 light).

**Figure 14:**
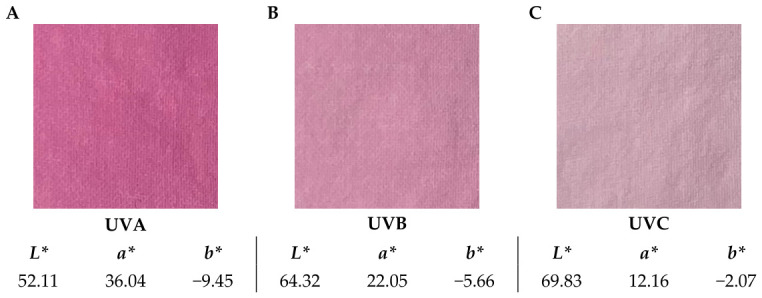
Comparison of wool samples printed with a paste containing 10% photochromic pigment after irradiation of 0.005 J/cm^2^ of (**A**) UVA, (**B**) UVB, and (**C**) UVC radiation with CIE L*a*b* values. The measurement error is 0.1%. All photographs were taken with a Canon 50D digital camera (kit lens: EF-S 18–200 mm f/3.5–5.6, resolution: 15.1 MPix; pixel size: 4.69 µm; standard D65 light).

Based on the chemical composition analysis, it was determined that the pigments would be applied to the surface of the textile material using the screen printing method. The use of printing paste ensured good binding of the pigment to the textile substrate while maintaining the elasticity and flexibility of the product. Woven wool fabric samples were printed with paste containing photochromic pigment according to the method described in Section 3.3. After drying, the samples coated with the paste layer were less yellow and brighter than the raw wool fabric sample (Figure 13A,B). This effect may result from filling the fabric structure with printing paste, which causes a different reflection of light from the surface of the textile product. The addition of pigment may also cause a change in the shade and luminosity of the sample because the photochromic pigment used for printing is a white powder without a distinct colour. As a result of UV irradiation, the white surface of the printed sample changes to an intense pink colour. Figure 13C shows the maximum intensity of the colour change after exposure to a dose of 0.5 J/cm^2^ of UVA radiation. The colour coordinate values in the CIE L*a*b* system are also provided below the sample photographs. Comparing the photographs, it can also be seen that covering the surface of the sample with printing paste changes the visibility of the weave of the wool fabric. Despite the plain weave used in the fabric and the slight difference in the number of threads in both thread systems, it seems that the paste reduces the visibility of the warp threads more. Therefore, the slightly thicker cross-section and less compact structure of the weft threads may cause greater water absorption, which may result in a slightly larger amount of printing paste being deposited on their surface. This aspect was not investigated in the further stages of the presented results because no other structures or textile raw materials were used to develop the printed UV sensor. To check the response of the printed wool samples to UV radiation, three samples were irradiated with a dose of 0.005 J/cm^2^ using UVA (315–400 nm; peak at 369 nm), UVB (280–360 nm; peak at 306 nm), and UVC (range: 100–280 nm; peak at 253.7 nm) cabinets. Upon UV irradiation, they all changed colours from white to pink, as shown in Figure 14.

**Figure 15:**
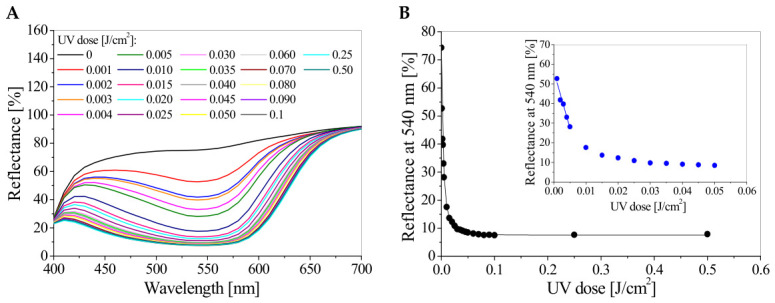
The reflectance spectra of wool fabric printed with paste containing photochromic pigment and irradiated with UVA radiation in the dose range of 0.001–0.5 J/cm^2^ (**A**) with the corresponded dose–responses with fitted curves for dynamic (third order exponential decay: y = 7.55 + 42.87e^−x/4.48×10^−3^^ + 14.73e^−x/4.64×10^−4^^ + 9.21e^−x/0.02^; R^2^ = 0.998) and linear (inset; y = −5783x + 56.42; R^2^ = 0.945) dose ranges (**B**). Measurements were performed immediately after irradiation. Each spectrum is the average of five measurements calculated by the measuring device with an error of 0.1%.

**Figure 16:**
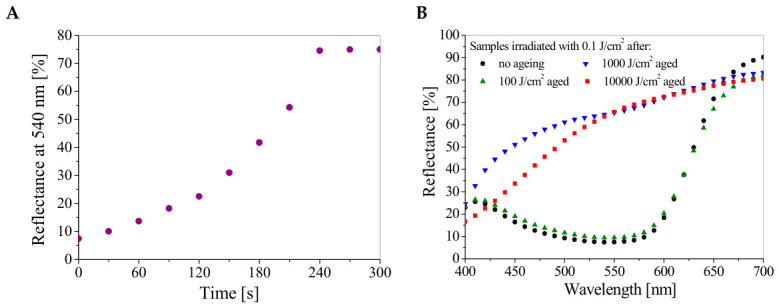
The light reflectance at 540 nm of a printed wool sample changed after five minutes of exposure to a UVA radiation dose of 0.5 J/cm^2^ (**A**) and comparison of changes in the reflectance spectrum of UVA radiation-aged samples (**B**).

The intensity of the colour depends on the dose and type of UV radiation. Comparing the samples, it can be seen that the greatest change in colour intensity occurs after irradiation of the sample with UVA radiation, which is also confirmed by the obtained results of CIE L*a*b* colour coordinate measurements. Thus, the samples irradiated with UVB and UVC have 23% and 34% lower colour intensity than the sample irradiated with UVA. Based on this observation and the fact that UV radiation reaching the Earth contains about 95% UVA and 5% UVB, only UVA radiation was selected for further studies. The studies also did not include a combination of UVA and UVB radiation to simulate the conditions of sunlight irradiation. Due to the rapid colour reversibility of printed samples after switching off the UV light and inserting them into the second chamber, the measurement uncertainty significantly impacts the obtained results. In the next step, the samples were irradiated with UVA radiation in the range of up to 0.5 J/cm^2^. For each sample, light reflectance was measured using a wavelength range of 400–700 nm (Figure 15A). The pink colour intensity of the sample increases with the radiation dose. The reflectance of the light decreases with the absorbed dose in the wavelength range of 500–600 nm, with a maximum at 540 nm. Thus, the calibration relationship in the dose range of 0.001–0.5 J/cm^2^ was determined and is presented in Figure 15B. The intensity of the pink colour reaches a plateau above 0.05 J/cm^2^. In the dose range of 0.1–0.5 J/cm^2^, no significant changes in reflectance were observed, indicating that saturation of the colour occurred. In addition, to characterise the response of printed wool samples to a UVA radiation dose, an equation describing the obtained calibration curve in the range of dynamic and linear dose–responses was determined. Dose–response equation with fitted curves for dynamic range of the UVA radiation dose ranges: y = 7.55 + 42.87e^−x/4.48×10^−3^^ + 14.73e^−x/4.64×10^−4^^ + 9.21e^−x/0.02^ (R^2^ = 0.998); linear range: y = −5783x + 56.42 (R^2^ = 0.945). Based on the above equations, the sensor parameters were characterised. The dynamic dose range of the printed sensors covers the region from 0.001 to 0.1 J/cm^2^, above which the system reaches a saturation plateau and no further colour changes occur. The sensor’s dose sensitivity corresponds directly to the slope of the linear regression curve. In the narrow linear range (up to 0.01 J/cm^2^), the sensor exhibits high dose sensitivity of −5783 ± 694%· cm^2^/J (*p*-value < 0.01) indicating a rapid decrease in reflectance at 540 nm per unit UVA dose. For the broader dynamic dose range (up to 0.1 J/cm^2^), the sensor’s response becomes non-linear and transitions into a saturation plateau, which is accurately described by the third-order exponential decay equation (adjusted R^2^ = 0.9980, reduced χ^2^ = 0.6357). The obtained results show that the developed printed sensors are capable of measuring the UVA radiation dose in the range of up to 0.1 J/cm^2^. The biggest problem in measuring the light reflectance of developed sensors is the time of holding the information about the absorbed radiation dose. Immediately after the end of irradiation, the obtained colour intensity of the sensor begins to decrease. In the case of other sensors based on textiles and radiation-sensitive precursors such as NBT, TTC, LCV, or LMG, the linear response to UVA and UVB radiation doses and the measurement range of these systems, depending on the development methods and concentration, are up to 0.1 and 1 J/cm^2^, respectively [25,26,27,28]. However, it should be emphasised that these systems are not reversible, and their selection and potential applications strictly depend on their sensitivity to the UV range and postradiation stability. Comparing the obtained results with other textile-based photochromic sensors poses a significant challenge due to the wide range of photochromic compounds used, differing in chemical structure and kinetic properties. The method of manufacturing such sensors and the textile substrate used can also lead to erroneous conclusions. It is worth emphasising that, although the general mechanism of this class of compounds’ response to UV radiation and visible light is known, commercially available solutions do not provide quantitative research results regarding their interaction with UVA and UVB radiation. Furthermore, no results were found regarding the pigment identified in this study, including the assessment of uniformity and the potential application of such modifications for monitoring two-dimensional UV radiation dose distribution. Figure 16A presents the change in light reflectance for the irradiated sample within five minutes after the end of irradiation with a dose of 0.5 J/cm^2^.

The obtained results show that the sample, after reaching maximum colour saturation, returns to white within four minutes. It has been shown that radiation-aged samples change their spectral characteristics. Increasing the radiation dose does not extend the time of retaining information about the colour intensity of the sample. Immediately after the irradiation process ends, the colour begins to bleach. The effect of sample ageing on the ability to continue irradiation and sensor stability was also examined. For this purpose, the samples were irradiated with high doses of UVA radiation above the saturation dose (100, 1000, and 10,000 J/cm^2^). After ageing, the samples were additionally irradiated with a dose of 0.1 J/cm^2^ to verify their continued functionality. Long-term exposure to UV radiation reduces the performance of the developed sensors, which results from the destruction of the chemical structure of photochromic pigment. For the sample aged with 100 J/cm^2^, after further irradiation with a dose of 0.1 J/cm^2^, a difference in the reflectance spectrum is visible, which is about 26% compared to the unaged sample (Figure 16B). In the case of samples aged with 1000 and 10,000 J/cm^2^, further irradiation was not possible because the samples were destroyed.

**Figure 17:**
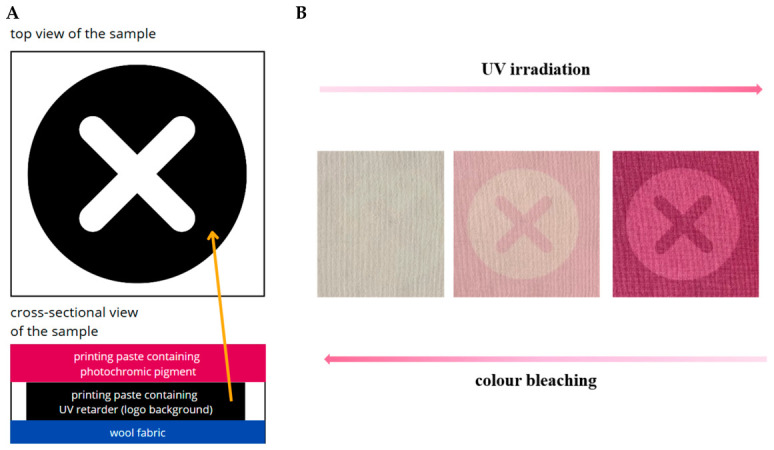
The scheme of printing (**A**) and the process of colour reversibility (**B**) of wool samples printed with a paste containing a photochromic pigment (10%) and a paste containing a UVA radiation retarder (20%) after irradiation with a maximum dose of 0.5 J/cm^2^.

To slow down the colour reaction during irradiation, samples containing the UVA retarder Rayosan^®^ C were also prepared, which was used to partially print the surface of the samples. Figure 17 shows the effect obtained after irradiating the samples with a dose of 0.5 J/cm^2^, necessary to obtain the maximum intensity of the sample. Unfortunately, this procedure did not affect the sample bleaching time after the irradiation process was completed.

**Figure 18:**
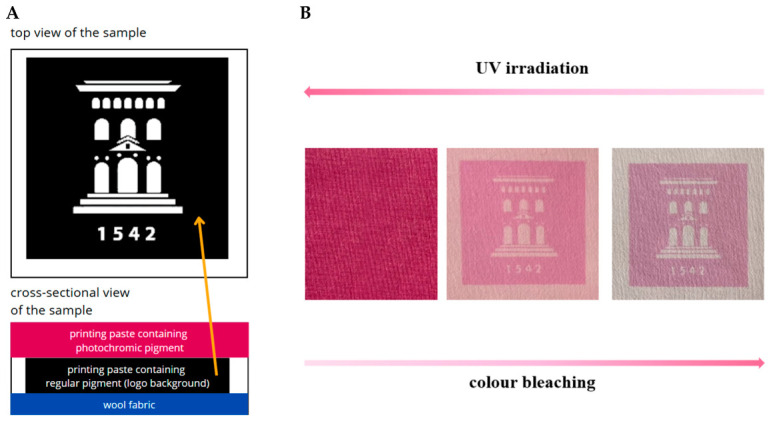
The scheme of printing (**A**) and the process of colour bleaching (**B**) of wool samples printed with a paste containing a photochromic pigment (10%) after UVA irradiation with a maximum dose of 0.5 J/cm^2^. Removing the source of UV radiation reveals a pattern made by screen printing using a standard pigment for dyeing printing pastes used in textiles.

Similar to samples without an additional printing layer containing a UV retarder, the sample turned white within five minutes after the end of irradiation. However, the obtained results may be intriguing from the point of view of designing sensors protecting textile products against counterfeiting. Using different concentrations of UV retarder, it is possible to produce printed areas with varying dose sensitivity. Figure 18 also presents the possibility of using photochromic pigment printing to cover patterns made using standard pigments for printing on the surface of textile products. Appropriate selection of the colour of the print made with the standard pigment and the colour intensity of the photochromic pigment layer after exposure to a specified dose of radiation results in complete coverage of the pattern. A properly designed pattern, in addition to providing information about the absorbed dose, can also act as a hidden security feature for textile or paper products. Such security sensors are not very popular in the textile production sector but are known for securing banknotes, papers, and packaging, e.g., medicines.

### 2.4. Measurement of UV Radiation Dose Distribution

**Figure 19:**
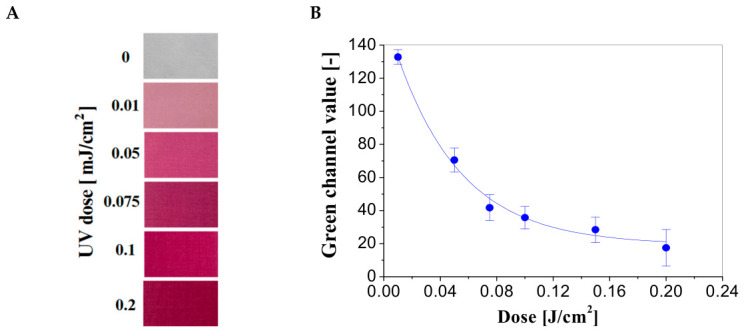
Image of scanned samples irradiated with UVA in the dose range of 0–0.2 J/cm^2^ (**A**) and calibration relationship of the green channel values for RGB colour model with respect to UVA dose absorbed by the samples (**B**).

To analyse the possibility of measuring the UVA dose distribution of the developed sensor, calibration samples were prepared, irradiated in the range of 0–0.5 J/cm^2^ and scanned using a flatbed scanner. Images of irradiated wool fabric samples after scanning are shown in Figure 19A. The visible structure of the fabric impacts the scanning of the samples, which was analysed in previous studies [29,48]. It is known that the parameters characterising the developed dosimeters for UV radiation measurements are greatly influenced by, among others, the type of raw material, fabric weave, scanner control software settings, scanning resolution, and data averaging after scanning.

**Figure 20:**
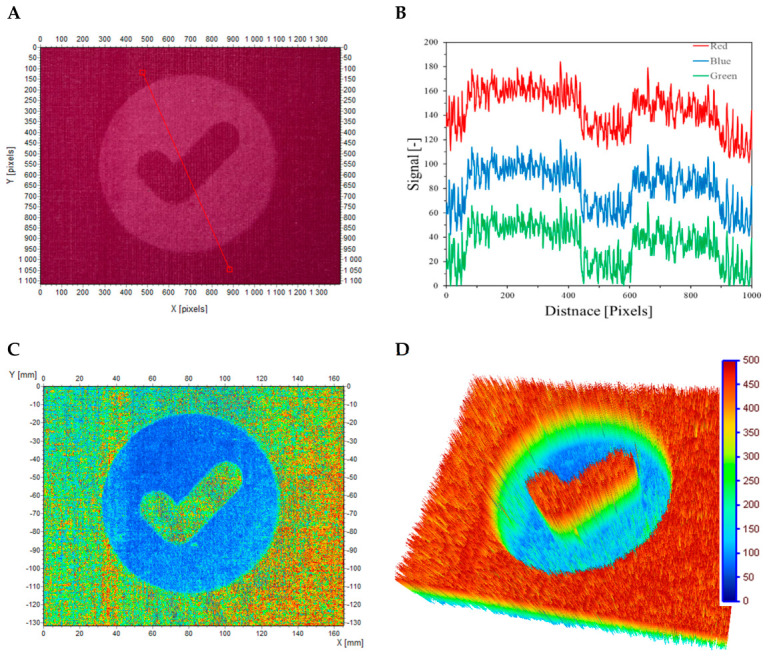
Results for a non-homogeneously irradiated sensor sample (UVA emitted dose of 0.5 J/cm^2^). (**A**) Scan of the sample after irradiation. (**B**) Change in colour intensity values in the three RGB channels. (**C**) A 2D absorbed dose distribution map. (**D**) The same 2D dose map illustrated in 3D with the dose scale (mJ/cm^2^) using the Plane 3D option of the polyGeVero^®^ software package (version 1.2). Dose maps were calculated using the calibration relationship given in Figure 19B.

To improve the quality of the obtained image, the samples were scanned at 75 dpi, and no sharpening or colour adjustment filters were applied. The sample images after scanning were decomposed into colour channels in the RGB colour model. Based on the comparison of the values from the red, green, and blue channels, it was decided that the green channel should be selected for further analysis. The calibration sample images (green channel) were prepared for the following settings of image processing: kernel size: 3 mm, kernel mode: 2D, iterations: 2, mean filter. A calibration relation between the values of the green channel of the RGB colour model and the absorbed dose was prepared, which is presented in Figure 19B. Each measurement point in Figure 19B is the average value from the image of the entire sample, an area of 7000–10,000 points with standard deviation bars marked. Thus, the calibration equation of second-order exponential decay was determined: y = 6.06e^−x/49.72^ + 2.31e^−x/44.09^ + 18.08 (R^2^ = 0.982). Analysing the response of the developed 2D sensor, the following conclusions can be drawn: (i) the dynamic dose–response is up to about 0.2 J/cm^2^, (ii) the decrease in the green channel value as a function of the absorbed dose can be described by the calibration equation, (iii) the dose sensitivity decreases with increasing pink colour intensity. Samples of sensors printed with photochromic dye are stable. However, the biggest disadvantage is their dynamic loss of dose information. Selecting conditions that slow down the photochromic reaction would also allow the use of the developed sensors for monitoring the dose distribution over a longer period of time, which was not assessed in this study. In the next step, the scanned image of the non-homogeneously irradiated sample (0.5 J/cm^2^) was converted into RGB channels, and the green channel values were converted into dose values after applying the calibration relationship (Figure 20A,B). As a result of this operation, a two-dimensional dose distribution map of UVA radiation was produced, as shown in Figure 20C,D. The obtained results show that the maximum recorded radiation dose is about 0.5 J/cm^2^. The obtained results match the actual dose emitted by the UV radiator used in the study and confirm the possibility of using the printed wool sensor for radiation measurements.

### 2.5. The Surface Unevenness Analysis of Printed Wool Samples—RGBreader Script and SEM

**Figure 21:**
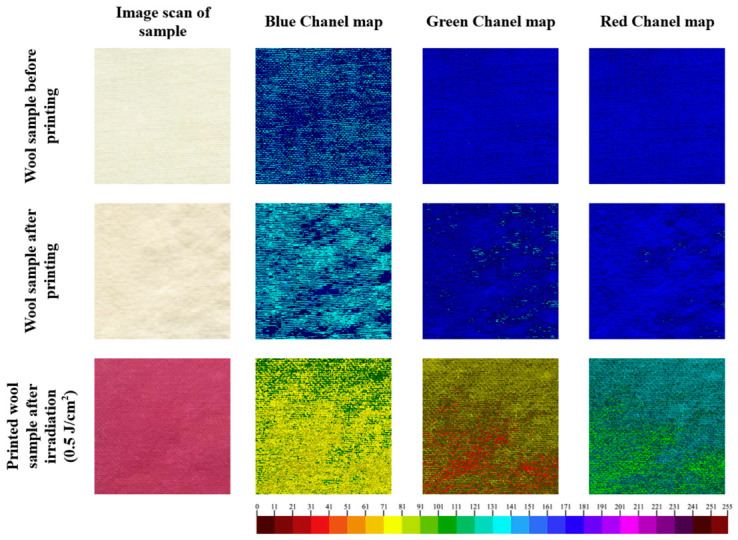
The macro photographs of wool samples and images after analysis of the unevenness of the fabric surface structure using RGBreader. Colour scale utilised by an algorithm for the analysis of tinge distribution on the surface of textile samples (0: ideal black; 255: ideal white).

In the presented research, wool fabric with a simple plain weave was selected, without a visible structure of weft and warp threads. This structure ensures less uneven distribution of the printing paste on the surface of the textile material. Samples of printed wool were scanned and analysed for RGB to determine the heterogeneity of the distribution of printing paste and photochromic pigment on the fabric surface. For this purpose, the image obtained after scanning with an Epson Perfection V750 Pro scanner was divided into three colour channels: red, green, and blue in the RGBreader Script as described in Section 3.5. Maps of surface unevenness distribution were made for each sample (Figure 21).

**Figure 22:**
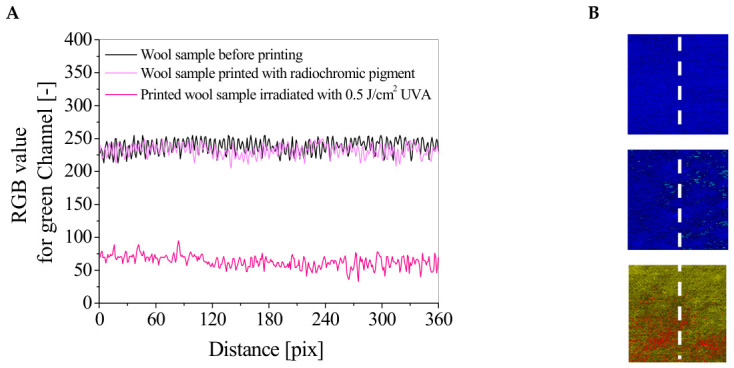
The green RGB channel profiles (1 pix = 0.1 mm) for non-printed, printed with paste with photochromic dye, as well as printed and irradiated with 0.5 J/cm^2^ UVA wool samples (**A**), together with the images of the RGBreader green channels of samples with white dash lines to indicate the position of the profiles (from the top: non-printed, printed, and printed and irradiated sample) (**B**).

**Figure 23:**
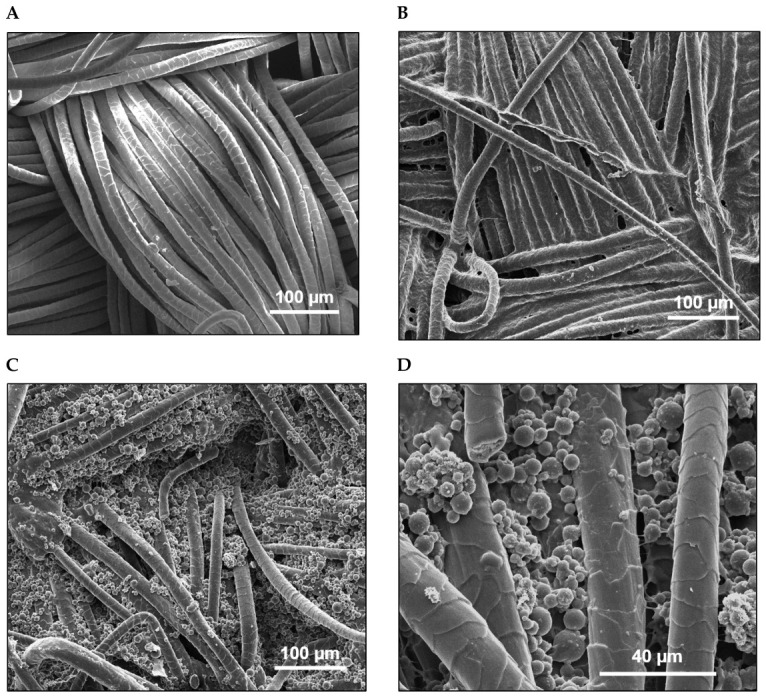
SEM images of the textile alone at 600× magnification (**A**), textile with printing paste at 600× magnification (**B**), and textile with printing paste and photochromic pigment at 600× magnification (C) and 2400× magnification (D).

**Figure 24:**
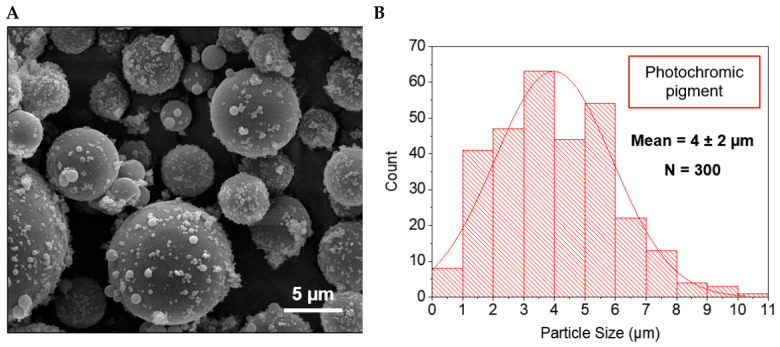
SEM images of photochromic pigment at 2400× magnification, (**A**) 10,000× magnification, and (**B**) size distribution histograms of the photochromic pigment.

To objectively determine the unevenness of the sample surface after UVA irradiation, the green channel was selected, for which the largest changes in value were observed from 0 to 255 in the RGB scale. Comparing the samples printed before and after irradiation with a dose of 0.5 J/cm^2^, slight irregularities on the surface of the samples are visible. They are characteristic of textile products made of natural fibres, which are creased without additional stabilising treatment and ironing. These creases, however, do not affect the homogeneity of the photochromic dye distribution on the fabric surface. Additionally, a comparative analysis of the green RGB channel profiles of the wool samples before printing, after printing, and after irradiation with a dose of 0.5 J/cm^2^ was carried out, and the obtained results are presented in Figure 22. The results confirmed the earlier conclusion that printing a layer of paste containing photochromic pigment on wool fabric slightly improves the surface uniformity. After irradiation, no significant differences in the colour distribution on the sample surface were visible, and therefore it was assumed that the samples were homogeneous. The spaces between the weft and warp of the fabric are also filled with a paste (Figure 23A–C), but the printed layer did not significantly affect the grip and aesthetics of the fabric. However, it is also visible that the pigment particles are spherical in shape with varying diameter sizes (Figure 23D). Although the origin and molecular structure of the photochromic pigment could not be clearly determined, SEM analysis shows that the pigment may be encapsulated. The particles are round, but their size is varied (Figure 24A), and the average particle size is 4 ± 2 µm (Figure 24B). On the surface of the round particles, additional inclusions of a substance are also visible, which is probably a compound that limits the pigment from clumping.

### 2.6. Application Possibilities

**Figure 25:**
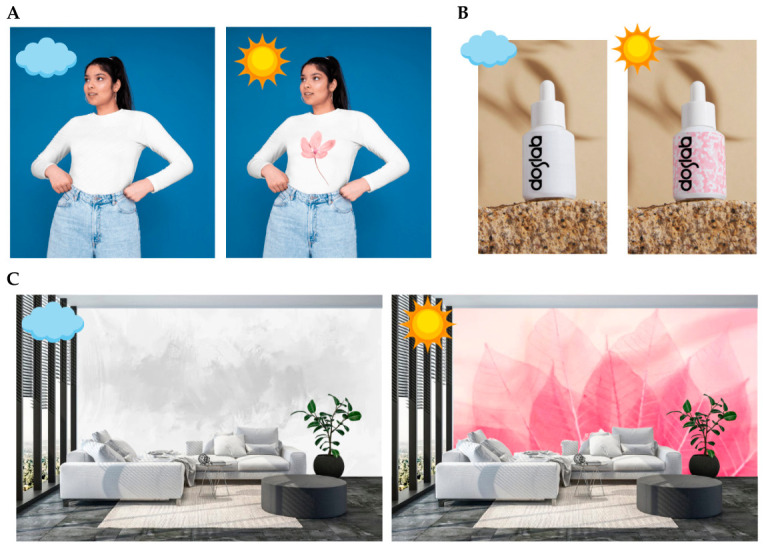
Example visualizations of the use of developed prints based on photochromic pigments in the clothing industry (**A**); packaging production (**B**); interior decoration as wallpaper (**C**).

Due to the dynamic pace of textile development, the solution proposed in this article, using photochromic pigments, can be a functional element of a wide range of technical, decorative, and personal products. The use of innovative solutions and the selection of materials are key to balancing the textile industry and attracting the attention of average users to the opportunity to invest in more interesting and durable products. In light of the new regulations regarding so-called “textile passports,” it is also important to provide information about the chemicals used to modify products. In the case of commercially available dyes and pigments, it is sometimes difficult to determine the origin and chemical structure of the compounds used in their production. Information provided by manufacturers or sales agents does not always provide complete information on the chemical structure of the compounds and is often inconsistent, for example, due to changes in trade names and the interchangeable use of the terms “dye” and “pigment” [2,3,4,12,49,50,51]. Dyes and pigments are essentially chemical colouring substances, but they differ in chemical structure, solubility, binding mechanism, and application possibilities. In the case of pigments, bath dyeing methods cannot be used because pigments are chemical compounds, usually inorganic, that do not dissolve in water or common solvents. They are typically produced in dispersions, which are used to colour polymers by volume, for example, in the production of dyed polyester fibres or in textile printing processes. Therefore, modifying the surface of textile products requires a medium that binds the pigment to the fibre. Two-component printing pastes are typically used for this purpose, consisting of a thickener and a binder, which are mixed with water in the appropriate proportion. After printing, drying and annealing are necessary to polymerise the print paste, which permanently bonds the pigment to the printed textile product. This type of textile surface finish is faster and easier to prepare. At the same time, selecting the right printing pastes ensures excellent pigment bonding with any textile substrate. A properly prepared sample also ensures separation of the functional layer from environmental factors and limits skin contact, for example, when such a component is used as protective clothing [23]. When using such printed sensors as components in clothing construction, selecting the appropriate paste is crucial, as it will ensure the durability of the functional finish throughout the product’s life cycle. In this study, the selection of printing paste components (Lutexal Hit and Helizarin Binder) was dictated by their established position as industry standards, known for ensuring high durability and flexibility of prints. These pastes, combined with other UV-sensitive compounds, have already been tested for resistance, including washing, as documented in previous studies [52,53]. The printing paste used was shown to have good adhesion to various fibres, including cellulose and polyamide, and to be wash-resistant for up to 50 cycles. Naturally, such materials should also be tested for their resistance to ageing, washing, and abrasion, but due to the complexity of these issues, they were not addressed in this work. With the appropriate selection of printing paste, it is also possible to print such sensors on other substrates, such as foils or paper, allowing for product marking in the packaging industry as well. Example applications of the developed sensors based on photochromic pigments are shown in Figure 25. Furthermore, such solutions can also be part of overt and covert security systems, which can be used to protect various products, including textiles and packaging, against counterfeiting. Thanks to the ability to monitor doses and two-dimensional UV radiation distribution, the developed sensors can also act as indicators of ageing or improper storage of products. Therefore, the possible applications could be much broader and include the production of personal protection sensors or structural components, including composites.

## 3. Materials and Methods

### 3.1. Analysis of the Chemical Structure of the Photochromic Pigment

Nuclear magnetic resonance spectroscopy (NMR): ^1^H NMR and ^13^C NMR, 2D HSQC, 2D HMBC and 2D COSY NMR spectra were recorded on an Avance Neo 400 NMR spectrometer (Bruker, Billerica, MA, USA; 400.15 MHz for ^1^H and 100.63 MHz for ^13^C, 9.4 T). NMR spectra were performed at room temperature for samples prepared by dissolving in deuterated acetonitrile (CD_3_CN). The NMR experiments were performed in CD_3_CN, as it provided sufficient solubility of the investigated photochromic pigment and ensured stable and well-resolved spectra without observable aggregation or precipitation.

Fourier-transform infrared spectroscopy (FTIR): FTIR spectra were collected at room temperature on a Nicolet 6700 spectrometer (Thermo Scientific, Waltham, MA, USA) equipped with a deuterated triglycine sulphate (DGTS) detector. The technique of attenuated total refraction (ATR) was used for measurements. The spectra were obtained by adding 128 scans at a resolution of 2 cm^−1^.

Differential scanning calorimetry (DSC): Thermal analysis of PLA samples was conducted by using a DSC TA Q20 analyser (TA Instruments, New Castle, DE, USA). The instrument was calibrated with temperature and heat flow using indium as standard. The samples (5–6 mg) were weighted accurately into hermetic aluminium pans and pressed slightly to ensure good contact with the DSC cell surface. The data were recorded during heating at a constant rate of 10 °C/min under nitrogen flow with an empty pan as the reference probe. 

The elemental analysis was performed with a CHNS analyser model 3018 from EuroVector s.p.a. (Milan, Italy). A sample, either analysed or standard, with a weight ranging from 1 to 5 mg and measured with an accuracy of 0.001 mg, was sealed in a tin capsule and loaded into the elemental analyser’s autosampler. The sample was then burnt in an automated process, and the resulting gaseous products were analysed chromatographically.

The surface atomic composition and chemical states of the pigments were measured by means of X-ray photoelectron spectroscopy (XPS), using a KRATOS AXIS ULTRA spectrometer equipped with a DLD analyser (Kratos Analytical, Tradfford, Manchester, UK). The X-ray radiation source was a monochromatic Al K⍺ (1486.74 eV) with a 120 W X-ray power and an anode voltage of 15.00 kV. The photo-excited electrons were analysed in constant pass energy mode, using pass energy of 160 eV for the survey spectra and 20 eV for the high-resolution core level spectra. CasaXPS 2.3.14 software (Teignmouth, Devon, UK) was used for data processing.

The morphological structure of the pigment was characterised by X-ray diffraction (XRD) on a PANalytical Empyrean diffractometer with a Cu K⍺ radiation (Malvern Panalytical, Almelo, The Netherlands). A quick wide 2θ range scan was performed (5–110°, 9.9°/min) as well as a slower scan of the area of interest (3–50°, 1.7°/min) to ensure that the structure did not change during the slow scan.

### 3.2. Preparation of Printing Paste

In this study, a wool fabric (bleached, without optical brighteners, plain weave, surface mass of 145 g/m^2^, thickness of 0.38 mm, weft setting of 260/dm, and warp setting of 270/dm, Tomtex S.A., Tomaszow Mazowiecki, Poland) was used. The printing process was carried out with an aqueous printing paste containing Helizarin Binder (BASF, Ludwigshafen, Germany), Lutexal Hit (BASF, Ludwigshafen, Germany), and photochromic pigment (PD-magenta, SFXC, Denton Island, UK). The printing paste was prepared as follows. Helizarin Binder (25 g) and Lutexal Hit (8 g) were mixed in 217 g distilled water. After 30 min of continuous mixing at a rotation speed of 1000 rpm, the printing paste was homogeneous and ready for use. The photochromic pigment was added to 300 g of the paste. The concentration of PD-magenta (K+L, Lodz, Poland) in the printing paste was 10% (*w*/*w*). All components were weighed on a laboratory balance with an accuracy of ±0.1 mg (model: AS220.X2 PLUS, RADWAG, Lodz, Poland). The selection of the paste component concentration made it possible to obtain a transparent layer on the surface of the printed textile material without a visible initial colour (all printed samples were white after printing, before UV exposure).

To test the possibility of delaying the reaction of the photochromic pigment colour change, some of the samples were additionally printed with a paste containing the UV radiation retarder Rayosan^®^ C (Archroma, Pratteln, Switzerland). For this purpose, 20 g of the retarder was added to the 100 g of printing paste obtained by mixing Helizarin Binder, Lutexal Hit, and water. After mixing the ingredients, the paste was used for printing the wool samples previously printed with the paste with the photochromic pigment.

### 3.3. Screen Printing

The screen printing method was used in the presented study. This method is simple, economical, and can be used for printing on the surface of various materials such as textiles, paper, plastics, ceramics, glass, and even metals. A stencil made from polyester mesh stretched on an aluminium frame (EX 63-063/160 PW screen: 43 mesh/cm; NBC, Tokyo, Japan; distributed by K+L Company, Lodz, Poland) and a photopolymerizing emulsion (Fotocoat 1010; Forteco, Kwintsheul, The Netherlands) were used to prepare the printing screen. The screen mesh was coated with a photopolymerizing emulsion and dried in a dark cabinet at 30 °C (5 h). After drying, the screen was ready to be exposed to UV light and to create a pattern. A simple pattern, squares (50 mm × 50 mm), was designed, which was created as a graphic digital file and printed on transparent foil. Then the foil with the black printed pattern was placed on the prepared screen and irradiated using a light frame (20 min; halogen lamp, Halogenfluter 500 W 930037; Düwi GmbH, Breckerfeld, Germany). After exposure, the screen was washed and dried. Then, the wool fabric samples were printed with a printing paste prepared as described in Section 3.1. The printing paste was applied to the fabric surface by pressing the paste through the screen using a soft rubber squeegee. Then, the printed samples were dried in a dryer (Binder FD23, BINDER GmbH, Tuttlingen, Germany) at 80 °C, with forced convection for 30 min. Based on the weight of 10 samples, it was determined that the paste collection using 1 g of wool sample was equal to 0.2017 g. After drying, the samples were covered with aluminium foil and protected from light. The packaging protected the printed samples from accidental irradiation. The prepared samples were ready for UV irradiation.

### 3.4. Irradiation of Samples

The printed wool fabric samples were irradiated in UV crosslinker chambers (UVP, UK) at three wavelengths corresponding to UVA (five lamps type F8T5 Blacklight, 8W, range: 315–400 nm; peak at 369 nm, Hitachi, Tokyo, Japan), UVB (five lamps type G8T5E, 8W, range: 280–360 nm; peak at 306 nm, Sankyo Denki, Tokyo, Japan), and UVC (five lamps type G8T5, 8W, range: 100–280 nm; peak at 253.7 nm, Sankyo Denki, Tokyo, Japan). The UV crosslinkers used for irradiation do not have the ability to change the angle of the UV light source in relation to the irradiated sample. Therefore, all samples were positioned perpendicular to the axis of UV light emission. Due to the reversible nature of the colour changes caused by the photochromic pigment used for printing, the samples were not fractionally irradiated. No combination of UVA and UVB radiation was used to simulate the conditions of sunlight irradiation. The given UV radiation dose was emitted by the crosslinkers automatically. The devices have a built-in detector and device control system (for example, the emission time of 0.1 J/cm^2^ UVA was 57 s). To check and confirm the emitted dose, an external UV radiation detector was additionally used. For example, for UVA radiation, the results obtained from measurements with the Lutron UV-340A detector (Lutron Instruments, Coopersburg, PA, USA) covered with a fixed dose emitted by the UV crosslinker, e.g., for a dose of 0.5 J/cm^2^, the average of 10 measurements was 0.48 ± 0.02 J/cm^2^. Therefore, the UV dose set on the irradiator (J/cm^2^) and measured by the irradiator’s internal detector was confirmed and is treated as the dose to which a sample was exposed to. All samples were irradiated in the dose range of 0.001–0.1 J/cm^2^.

### 3.5. Reflectance of Light Measurements

The reflectance spectra of the wool woven fabric samples printed with photochromic pigment paste were measured with a Spectraflash 300 light reflectance instrument (xenon lamp D65; angle 10°; 10 nm resolution; the measurement error is 0.1%; DataColor, Rotkreuz, Switzerland). Before the measurements, the device was calibrated as described elsewhere [28]. UV light in the wavelength range of 190–400 nm was automatically cut off by the software (microMATCH v. 3.6; DataColor, Rotkreuz, Switzerland) when the 0% UV option was selected to avoid unnecessary irradiation of the samples. The wool printed samples were measured immediately after irradiation and over time after irradiation over the wavelength range 400–700 nm. The wavelength at which the change in light reflectance was maximum was then selected and discussed in relation to the absorbed UV dose. Based on the measurements, the characteristic parameters of the sensors were determined. In addition, the colour coordinates were determined with the CIE Lab colour system, which describes the perceived colour according to the standard ISO/CIE 11664-4 [54].

### 3.6. Surface Analysis and Evaluation of the Surface Unevenness

The morphology of the raw wool sample and printed wool samples was analysed using a field-emission scanning electron microscopy (SEM, FEI Inspect F50, Thermo Fisher Scientific, Hillsboro, OR, USA) with a typical acceleration voltage of 10 kV. The samples were deposited on carbon conductive tape and metallized with palladium. The particle size was measured from these images using ImageJ software (version 1.54 ) Additionally, compositional analysis was performed using energy-dispersive X-ray (EDX) spectroscopy at an accelerating voltage of 20 kV and a working distance of approximately 10 mm. For the EDX analysis, the samples were coated with a carbon layer to ensure optimal signal detection.

To determine the unevenness of the unprinted and printed wool surface, the samples were scanned with an Epson Perfection V750 Pro scanner (cold cathode fluorescent lamp; optical resolution Main 6400 DPI × Sub 9600 dpi; 48 bits/colour; Epson, Nagano, Japan). Samples with dimensions of 36 × 36 mm^2^ were scanned with the following parameters: (i) resolution of 300 dpi; (ii) reflection mode with 24-bit RGB colour depth; and (iii) scanning parameters (brightness, colour correction, and sharpness) were disabled and were not considered in this study. Based on the obtained scans, maps of surface unevenness distribution were made, and sample profiles were calculated using a prepared script for reading the RGB channels (RGBreader; Python Script 3.9 with Python Imaging Library; DosLab [29]). Each sample was imaged using a three-colour RGB scale (red, green, and blue). After the initial analysis of the tested samples, the green channel was selected for all samples for which the largest changes in value were observed from 0 to 255 in the RGB scale.

### 3.7. Dose Distribution Assessment

The printed wool samples were also subjected to UV radiation dose distribution testing. For this purpose, the printed samples, irradiated in the range of up to 0.1 J/cm^2^, were photographed using a Canon 50D digital camera (Canon, Ota, Japan, RGB mode, resolution 15.1 MPix, pixel size 4.69 µm, without flash, no automatic colour correction) equipped with the kit lens (EF-S 18–200 mm f/3.5–5.6) in standard D65 light. Due to the rapid reversible colour change, all samples were scanned immediately after exposure. To avoid accidental exposure to sunlight or artificial light, measurements were taken in a darkened room.

The obtained sample photographs were processed using the polyGeVero^®^-CT software package (v.1.2, GeVero Co., Lodz, Poland) [30]. Based on this, the sample calibration equation was developed and used to convert the photographs into 2D dose distribution images. Dose maps were also exported to the polyGeVero^®^ software package (v.2.0, GeVero Co., Lodz, Poland) for further analysis. Based on the photographs of wool samples printed with photochromic dye, the following was done using the software package: (i) calibration of the samples, including calculation of the calibration equation; (ii) conversion of the obtained results into UV dose distribution; and (iii) creation of 2D/3D maps with UV dose distribution.

### 3.8. Cytotoxicity Assessment

The cytotoxicity evaluation was conducted in accordance with the ISO 10993-5 standard [46]. Primary human dermal fibroblasts (hDF, PELOBiotech GmbH, Planegg, Germany) were cultured in Fibroblast Basal Medium without glutamine (PELOBiotech GmbH, Planegg, Germany), supplemented with the Fibroblast Growth Medium Kit Classic (PELOBiotech GmbH, Planegg, Germany) and 1% penicillin–streptomycin (Biowest, Nuaille, France). Cells were maintained at 37 °C in a humidified atmosphere containing 5% CO_2_. For cytotoxicity testing, cells were seeded in 96-well plates at a density of 6 × 10^3^ cells per well and incubated for 24 h. Subsequently, the culture medium was replaced with 100 µL of test samples. Untreated cells served as the negative control, while cells treated with Triton X-100 (Sigma-Aldrich, Saint Louis, MI, USA) at a final concentration of 0.01% were used as the positive control. Two types of test samples were prepared by suspensions of photochromic pigment and eluates of textile materials: (i) photochromic pigment (0.1 g) was dispersed in 10 mL of culture medium (1% *w*/*v*), incubated for 24 h at 37 °C with agitation, and then diluted serially to concentrations ranging from 0.1% to 0.00001%, and (ii) rectangular fragments (1 × 4.5 cm) of textile samples, including raw wool, wool printed with plain paste, and wool printed with photochromic pigment were incubated in supplemented culture medium at a ratio of 3 cm^2^/mL for 24 h at 37 °C under continuous agitation, in accordance with the ISO 10993-12 standard [47]. Eluates were sterile-filtered (0.2 µm) and tested at concentrations of 100%, 50%, and 25%. After 48 h of exposure to the test samples, the medium was replaced with 100 µL of fresh medium containing Thiazolyl Blue Tetrazolium Bromide (MTT reagent, Sigma-Aldrich, Saint Louis, MI, USA) at a dose of 1 mg/well. Plates were incubated for 4 h at 37 °C, after which the supernatants were removed, and the resulting formazan crystals were dissolved in 100 µL of 70% isopropanol containing HCl. Absorbance was measured at 570 nm with a reference wavelength of 630 nm using a BioTek microplate reader (CTL, Winooski, VT, USA). Cell viability was calculated as a percentage relative to the untreated control. Data are presented as the mean ± standard error of the mean from three independent biological replicates, each performed with eight technical replicates (*n* = 8).

## 4. Conclusions

This work presents the development of a printed photochromic sensor for marking and securing textile products. The proposed method is fast, simple, and relatively cheap. The printing paste containing a photochromic pigment applied to the textile product using a screen-printing method does not require the use of specialist equipment and is a commercially available technique used in the production of textiles. The developed sensor changes colours from white to pink under the influence of UVA radiation. The colour intensity increases with the increase in radiation exposure. This effect is reversible, which allows for multiple uses of the sensor. A detailed characteristic of the sensor’s response to the dose of UVA radiation is presented. The sensor works in the linear dose range of 0–0.005 J/cm^2^ and in the dynamic range for doses of 0–0.05 J/cm^2^. In the conducted chemical tests, the characteristics of a commercial photochromic pigment were determined. The data obtained from chemical analysis contributed to the proposed structural interpretation of the photochromic pigment, ethyl-3′-methyl-3′-phenyl-1′-(propan-2-yl)-1′,3′-dihydrospiro[[4,1,2]benzoxadiazine-3,2′-indole], including insights into aromatic substitution patterns and the presence of functional groups. Due to the minimal amount of information about the purchased pigment, tests of its elemental composition and cytotoxicity tests on human dermal fibroblasts were also carried out. It has been shown that, at high concentrations, the pigment can have toxic effects on skin cells, potentially through direct chemical interaction or poor dispersion stability. However, using the pigment in printing pastes, its possible toxic and skin irritation activity may be significantly reduced. On this basis, it was confirmed that the developed sensors are safe for external use as printed layers. In addition, the possibility of using it to read the dose distribution in 2D was demonstrated. With the right choice of measuring devices or a developed reference colour scale, such sensors can be used to read UVA radiation doses. Thus, it can be used as an element confirming the authenticity of a textile product, securing the supply chain, or as a decorative element. The conducted research does not exhaust the issues related to the methods of securing and marking textile products. It is intriguing to develop sensors that would respond in a hidden and certified way only to a specific wavelength of radiation.

## Data Availability

The data supporting the reported results are not stored in any publicly archived datasets. The readers can contact the corresponding author for any further clarification of the results obtained.
